# Everybody's Page

**Published:** 1890-03-29

**Authors:** 


### Everybody's Page.

Modern Rome is said to be the city best supplied with water in the world, but Ancient Rome had a supply of nearly seven times the quantity.

Sir Crichton Brown says that on the average an Englishman's head weighs an ounce less than a Scotchman's, a Scotch head 50 oz., an English 49 oz. We are not jealous. Weight is not everything.

It has been said that M. Miguel, in his experiments on the milk of a cow, found in it 19,320 bacteria per cubic centimetre, and that these, kept at a temperature of 25 deg. C., increased in 21 hours to the number of 200,000,000 per cubic centimetre.

We who revel in cheap editions have a great deal to be thankful for, for the care books were taken of in their infancy must have been rather a bore. The Romans used to shut up their roll-books in cedar boxes, so that the smell of the wood might keep off moths and other searchers after knowledge. Now to undo a little box whenever we wanted to refresh our memory would scarcely suit nineteenth century temperaments.

For thoroughgoing brutality the Russian official has yet to find his equal, and that should not, it is to be hoped, be accomplished with ease. The Bulgarian atrocities pale by the side of the alleged treatment of Siberian prisoners. Inhumanity is too good a word to apply to the fiendish acts of some so-called men. No retribution, and the day of retribution must come, will be too severe for the ruling classes in that unhappy country.

Agrarian troubles do not belong exclusively to England and Ireland, but Japan comes in for her share. The kind-hearted considerate landlord is almost extinct, and money-lenders from the towns are possessing the land. The Japanese pay their rent mostly in certain measures of rice, quite regardless of market value or the state of harvest, so when rice is scarce and dear this is not very satisfactory, and the cry is being raised by the tillers of the land for laws to improve their lot.

With regard to our recent paragraph on the Wild Birds Protection Act, the Rev. F. O. Morris sends us the following note from Lord Hope, which was addressed to the editor of the *Daily Graphic*. It shows that the clause regarding birds' eggs is a most necessary one : "Sir,—I have read your comments upon Mr. F. O. Morris's agitation for the protection of birds and their nests. As a lover of birds I do all in my power to protect them. Allow me to point out that it is not boys only who rob the birds of their eggs. In Sussex the men employed on the farms and gardens take every egg they can find, and employ their dinner hour in searching for and eating them. Even gamekeepers are guilty of this. On my own place in Sussex, last year, I gave an employment to an extra man, strictly cautioning him against touching the birds' nests, of which there were a great many. To my surprise, I soon found that nearly every nest had been robbed of its eggs. I challenged the man, when he said, 'Yaas, I took 'un.' 'And what did you do with them?' said I. 'I ate 'un ; I allus allows to," said he. I packed him off at once. The labouring men destroy more than the boys. The goldfinch was very common here ten years ago ; now it is very scarce. The birds are either taken as nestlings or as soon as they can feed well, and are used as decoys for the old birds. Surely it is possible to punish those who are guilty of the wholesale destruction of our feathered songsters. Yours obediently, Hope. Horsham, Sussex, Febuary 7th, 1890."

The French word "microbe" was originally introduced to overcome the difficulty of deciding whether these low organisms were plants or animals.

The other day we overheard a gallant 'bus driver say to the conductor, as some of the fair sex mounted to the garden-seats, "The ladies are my barometer. I always know it's going to be fine weather when the ladies come up on top."

Where do all the stray dogs come from? There seem to be plenty of them, "both mongrel, puppy, whelp, and hound, and cur of low degree." Perhaps some of them are unsuspecting country cousins; anyhow the canine race is not good at avoiding the arm of the law, for 7,000 doggies are said to have been conveyed to Battersea this year.

The gold sand of the Russian mines of Yakutsk is said to yield less gold than in the past, but as the discovery of gold in quartz has risen to twice its former weight it is not of much consequence. Here is a country with plenty of gold, and they cannot get enough labour to mine it, for the reason that there is no railway to convey the labourers to the gold fields.

There are a great many delicate (?) members of society who will assure you in pathetic tones that they "can't live on clay," or "must live on chalk," but, oddly enough, these very persons are generally those who possess a sufficient quantity of the almighty dollar to enable them to live where they like. Yet it is astonishing how well some of us less fortunate people keep, who have to live where rents suit our pockets, and not where soil suits our system.

Of all the awful accidents which occur daily in a large city like ours, perhaps burning is the most alarming. It is well to remember that in severe cases the shock due to the fright as well as the burn is the first thing to be considered. If medical help is not obtainable at once the best thing to do is to wrap the burnt person in a blanket, put him in a warm place with hot bottles to his feet, and give him a little hot brandy and water, or something of that sort. The easiest applications to procure in an emergency with which to cover up the wounded parts from the air are flour or salad oil.

There is no end to the list of articles that can be produced for the large sum of a penny. Penny toys to begin with, which are so remarkably well turned out as to stimulate the British youth into making small collections of them in order to show what can be obtained for a penny, though personally we fear that an inquiry into the wages of those who fashioned these toys would rather detract from our admiration of them. Then there are penny packets of soup, which are not by any means to be despised; and lastly, we hear of the "penny combination tea ball," containing infusions for two cups of strong tea, sugared and creamed! So evidently the penny delectable delicacy will be the marvel of the hour.

That was a very pretty story which the *Daily News* related the other day, telling how Dom Pedro of Brazil not only managed to give Rio a splendid hospital, but how he also managed to rebuke human vanity. Dom Pedro saw the necessity for a hospital, but he could not afford to build one, nor could he induce the rich to subscribe. But inspiration came to him. Anybody who subscribed largely to the hospital was to have the right to call himself by some title—count or baron—for his lifetime only. And as "all is vanity" the money rolled in and the hospital rose, and the Latin motto which the Emperor placed over its gates was "Human vanity to human misery."

